# Bioinformatics for wet-lab scientists: practical application in sequencing analysis

**DOI:** 10.1186/s12864-023-09454-7

**Published:** 2023-07-07

**Authors:** Vera Laub, Kavi Devraj, Lena Elias, Dorothea Schulte

**Affiliations:** 1 Neurological Institute (Edinger Institute), University Hospital Frankfurt, Goethe University, Frankfurt, Germany; 2 Department of Biological Sciences, Birla Institute of Technology and Science Pilani, Hyderabad Campus, Hyderabad, Telangana India

**Keywords:** ChIP-seq, RNA-seq, ATAC-seq, Integrated data analysis, Transcriptional networks

## Abstract

**Background:**

Genomics data is available to the scientific community after publication of research projects and can be investigated for a multitude of research questions. However, in many cases deposited data is only assessed and used for the initial publication, resulting in valuable resources not being exploited to their full depth.

**Main:**

A likely reason for this is that many wetlab-based researchers are not formally trained to apply bioinformatic tools and may therefore assume that they lack the necessary experience to do so themselves. In this article, we present a series of freely available, predominantly web-based platforms and bioinformatic tools that can be combined in analysis pipelines to interrogate different types of next-generation sequencing data. Additionally to the presented exemplary route, we also list a number of alternative tools that can be combined in a mix-and-match fashion. We place special emphasis on tools that can be followed and used correctly without extensive prior knowledge in programming. Such analysis pipelines can be applied to existing data downloaded from the public domain or be compared to the results of own experiments.

**Conclusion:**

Integrating transcription factor binding to chromatin (ChIP-seq) with transcriptional output (RNA-seq) and chromatin accessibility (ATAC-seq) can not only assist to form a deeper understanding of the molecular interactions underlying transcriptional regulation but will also help establishing new hypotheses and pre-testing them in silico.

## Background

**Fig. 1:**
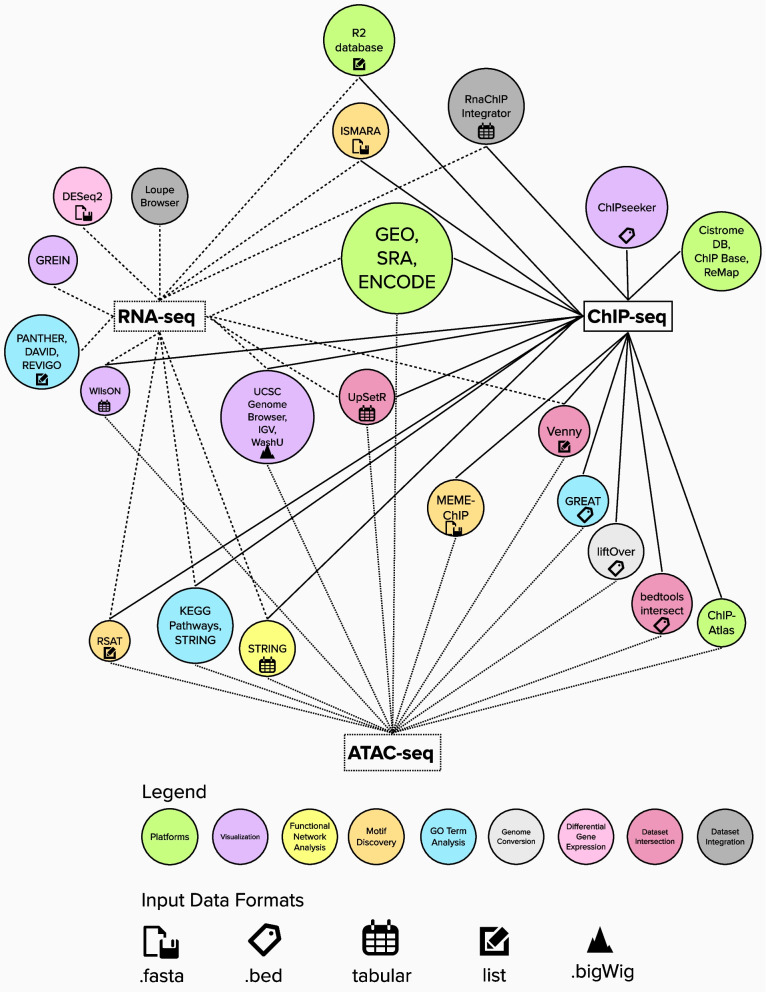
Tools and platforms presented for NGS data retrieval and analysis. The type of analysis is depicted by the color of circles, input data formats are given by pinned icons. Lines connect each NGS paradigm with bioinformatic resources applicable to this data type

In recent years, a plethora of methods were established in genomics research, approaching the question of what constitutes organisms on most basic levels from a variety of angles. Many of these methods make use of high-throughput sequencing to address gene expression at multiple levels, ranging from transcription (investigated e.g. by RNA-seq [1]) over accessibility of chromatin (assessed e.g. by ATAC-seq [2]) to the epigenetic modification of chromatin and site-specific binding of proteins to DNA, examined by methods such as chromatin-immunoprecipitation followed by massive parallel sequencing (ChIP-seq [3]). Lowering the cost of sequencing experiments has resulted in an abundance of genomic data available in the public domain. In the Bioinformatics community, this data has long served as resource for the development of analytical tools, while molecular laboratory scientists are just beginning to explore material that was published as part of a research project other than their own (for example see [4]). In addition to historic and epistemic differences between these two scientific cultures [5], we suspect that this effect is, at least partially, also rooted in a certain lack of accessible resources and training available to bench scientists. Here, we present an analysis pipeline that makes use of different platforms to retrieve sequencing data from the public domain together with freely available, user-friendly and predominantly web-based bioinformatic tools for the evaluation and visualization of results. As many platforms and tools can be used for several sequencing paradigms, visualized in Fig. 1, and to make the content most accessible to new users, specific aspects of their application will be introduced in different subchapters of this paper. The initial bioinformatic analysis of raw next-generation sequencing (NGS) results, including quality filtering, alignment of the reads to the genome and peak calling, is not subject in this review article but should follow appropriate guidelines such as those curated by the ENCODE Consortium. We here focus on the type of analysis that builds on already-processed datasets, enabling analysis steps such as comparison between datasets, mapping of individual data points across datasets, searching for gene ontology terms, jointly regulated pathways, or shared upstream regulators, and more. We present one exemplary route and follow it throughout the paper but suggest alternative tools and approaches alongside. This allows users to develop an analysis strategy that fits their needs and matches their preferences. We argue that this approach can serve as a valuable resource to explore new ideas and projects in silico, before moving forward with time-, cost-, and resource-intensive wet-lab experiments. Data resources are continuously growing and the here described databases are frequently augmented with new datasets. Nevertheless, data on many target genes or cell types are still missing from these repositories. New wetbench experiments will therefore be surely needed for the foreseeable future. Making the resulting data openly accessible is thereby a critical and valuable contribution to the scientific community.

## Data storage and accession

Most scientific journals require that all sequencing data submitted as evidence in a particular study are being made available in a public repository after the manuscript has been accepted for publication. With the publication, an accession code is provided to retrieve the data from respective platforms. Several options are available for this. For bottom-up approaches, these platforms can be searched for available datasets in order to start a new research project from previously published data. Own sequencing results can also be included and combined with public datasets. Commonly used platforms described in detail here are listed in Table 1.

**Table 1:** Data resources

Purpose	Platform/Software	Features	References
ChIP-seq data	ChIP-ATLAS	Data can be downloaded in *.bed* and *.bigWig* format;	[6, 7]
		Data can be readily visualized in IGV;	
		Peak calling differs from original publications	
	Cistrome DB	Information on QC; Motifs underlying peaks;	[8]
		Nexus to Galaxy analysis pipeline;	
		Human and mouse data only; Download of data in *.bed* only	
	ChIPBase	Transcriptional regulatory networks of lncRNAs, miRNAs, other ncRNAs	[9, 10]
		and protein-coding genes; Motif information included;	
		No raw data can be downloaded	
	ReMap	Collection of manually curated ChIP-seq, ChIP-exo, and DAP-seq data;	[11]
		Data can be directly accessed via UCSC Genome Browser	
Functional genomics	ArrayExpress	Includes metadata detailing experimental procedures	[12]
		as well as processed and/or raw data	
	ENCODE	Raw as well as processed data; Good QC and reproducibility	[13, 14]
General seq repositories	GEO Accession Viewer	Comprehensive study overview; Contact information to data curators;	[15]
		No consistent data formats available	
	SRA Run Browser	Hosts raw data files	[16]

Two frequently used platforms for data storage are Gene Expression Omnibus (GEO) for processed data and Sequence Read Archive (SRA) for raw data files. An alternative resource for functional genomics data is ArrayExpress [12]. It includes metadata detailing experimental procedures as well as processed and/or raw data. While ArrayExpress is hosted by the European Molecular Biology Laboratory-European Bioinformatics Institute (EMBL-EBI), which is part of the intergovernmental European organization ELIXIR, GEO and SRA are hosted by the US based National Center for Biotechnology Information (NCBI). Because these databases have been separately maintained, users might need to search several databases to get a comprehensive overview of public genomics data of interest [17]. Since errors can occur when uploading datasets as well as descriptive meta-data into databases, it may also be helpful to cross-check relevant information of individual datasets across platforms and in the original publication. GEO includes data from many different genetic and genomic approaches, including genome methylation, chromatin structure, and genome-protein interactions [18]. Each dataset on GEO, accessd via the GEO Accession Viewer, is provided with contact information of the researcher who generated it as well as a reference to the corresponding publication, if available. Datasets of multiple experiments in a given study (including different sequencing paradigms) are assembled in series that are linked on GEO Accession Viewer and can therefore be found with ease. While the provision of descriptive data regarding the experimental process applied to obtain a certain dataset is standardized on GEO Accession Viewer, the available datasets vary in format.

Another valuable resource is the Encyclopedia of DNA Elements (ENCODE) project. ENCODE collects a wealth of datasets from various sequencing paradigms, metadata as well as protocols and provides various data formats (both raw and processed) in order to systematically map regions of transcription, transcription factor (TF) association, chromatin structure and histone modification [13]. However, as compared to the platforms discussed above, the ENCODE project follows a specific scientific aim rather than providing a mere collection of data, and thus is focused on in-depth assessment of specific factors, rather than a wide range of transcriptional regulators. Therefore, most projects are centered around common cell lines and ubiquitously expressed factors or histone marks.

Of note, most datasets in the public domain arise from cell culture experiments, which are often chosen for their practical advantages in culturing, providing a homogeneous cell population. However, this comes at a cost in that cell lines are only an approximation of the primary cell types which are modeled. When making use of public datasets, potential (epi-)genetic differences that might be introduced should be critically assessed. Where available, it is further advisable to juxtapose this data with such from primary cells or tissues.

## Software repositories and general tools

Many bioinformatic tools as well as databases can be found on bio.tools, accessed via this registry and applied online or after downloading to a local computer. The code-base of tools is usually hosted on Github or Bioconductor. Github is an online platform that allows users to create repositories to store and share both, analysis code in all programming languages and datasets. A great many repositories can be created and openly shared using a free personal account, but contributors also have the option to restrict the use of their repository by a license. The website of the public repository can then be used for ease of code as well as data sharing in publications. As an example, the public repository created for analyzing data with UpSetR discussed in more detail below can be found here. For large datasets, file hosting services are recommended whose file links can be shared from the public folders for use in Github. Bioconductor is an initiative for the collaborative creation of bioinformatic software, harboring a multitude of open-source and open-development programs written in the statistical programming language R [19]. Commonly used tools described in detail in this paper are listed in Table 2 (ChIP-/ATAC-seq analysis) and Table 3 (RNA-seq analysis). This list could be extended much further as the demand for and development of sequencing analysis tools continuously grows, but we restrict ourselves to a selection of approaches that can be fitted into one exemplary pipeline.

**Table 2:** Platforms and tools for ChIP-/ATAC-seq analysis

Purpose	Platform/Software	Features	References
Annotation/ visualization	ChIPseeker	Available on Galaxy;	[20]
Assembly conversion	liftOver	Easy-to-use online version;	[21, 22]
		Only available for genomic loci conversion, not nomenclature	
General data analysis	Galaxy	Collection of bioinformatic tools;	[23, 24]
		Reproducible analysis pipelines	
Genome arithmetic	bedtools	Available on Galaxy;	[25]
		Easy-to-use terminal version	
GO term analysis	GREAT	Cis-regulatory regions supported	[26]
Motif discovery	MEME-ChIP	Available on Galaxy;	[27]
	RSAT	Integrates more database options for motif discovery than MEME-ChIP;	[28]
		Includes original analysis, such as motif quality evaluation	
Raw data quality control	FastQC	Easy-to-use desktop version	[29]
Track visualization	UCSC Genome Browser	Abundance of integrated data;	[21, 30]
		Export of *.eps* graphics, can be converted to publication-quality	
		figures using appropriate software	
	IGV	Online and desktop version	[31]
		High quality resolution;	
		Limited integration of data from other	
		sources as compared to UCSC	
	WashU	Cistrome DB carries direct plugins	[32]

**Table 3:** Platforms and tools for RNA-seq analysis

Purpose	Platform/Software	Features	References
Dataset intersection	Venny	Easy application;	[33]
		Low image quality for download	
	UpSet	Enables complex comparison	[34]
Data visualization	WIlsON	Provides data visualization including PCA, heatmap and scatterplot;	[35]
		Requires CLARION file	
	GREIN	Provides data visualization including PCA plots, and heatmaps,	[36]
		2D and 3D tSNE;	
		Uses GEO IDs of existing public datasets as input	
Differential gene expression	DESeq2	Yields fold-changes and statistical significance	[37]
		for every expressed gene between the samples of interest;	
		Included as a part of many RNA-seq pipelines and platforms	
		(R2, WIlsON, GREIN etc.)	
	GEO2R	Direct application to GEO deposited data	[18, 38]
Functional analysis	Enrichr	Can be queried for any size of lists up to single genes;	[39]
		Provides information on consensus TFs, lncRNAs, epigenetic roadmaps	
		of histone marks and motif enrichment	
GO term analysis	PANTHER	Takes list of gene names as input (several IDs supported);	[40, 41]
		Can work with large number of different species;	
		Low image quality of produced plots	
	STRING		[42, 43]
	DAVID		[44, 45]
	KEGG PATHWAY		[46–48]
	REVIGO	Reduces functional redundancy of GO term lists, visualizes results	[49]
Nomenclature conversion	g:Convert	Available conversion between multiple namespaces and organisms	[50]
	BioTools.fr	Only most commonly used namespaces (UCSC ID, refSeq and	
		ENSEMBL Gene ID) available	
Transcriptional networks	ISMARA	Provides motif information on promotor area,	[51]
		but not cis-regulatory regions	
	RSAT *network-interactions*	Can be easily integrated with other RSAT tools	[28]
	STRING	Provides information of protein-protein interactions of gene products	[42, 43]
	oPOSSUM		[52]

### Tools for dataset conversion

Challenges that researchers often face when retrieving data from public repositories are the different file formats and annotations in which the data is stored. For example, ChIP-seq data can be deposited in a variety of formats ranging from raw data in *.fasta* or *.fastq*, over processed data in simple tabular, human-readable *.bed* files to continuous track formats such as *.bigWig*. While *.bed* formats contain the coordinates of sequencing peaks, and thus can be viewed as quantitative data structures providing information on the presence or absence of peaks [53], *.bigWig* is a more qualitative data structure that also enables the assessment of peak shapes. Complicating matters further, most downstream applications have precise data structure requirements (visualized in Fig. 1) that do not necessarily match the structure in which the corresponding data is stored in public repositories. However, many of these formats can be translated into one another. An easy to use resource for this purpose can be found in the Galaxy platform, a system for the integration of genomic sequences, their alignments, and functional annotation [23, 24]. For example, the UNIX command line application bedtools getfasta available on Galaxy allows the conversion of *.bed* data into the *.fasta* file format. In case of annotation differences between datasets, annotation transfer tools such as liftOver can convert genome coordinates of *.bed* files into the respective assembly [21]. This enables users to integrate data from different annotation generations of the same species (e.g. mm9 and mm10 when working with mouse-derived data) and thus to compare results that were mapped to different genomic assemblies. In addition, with the help of liftOver genomic annotations from a wide range of species can be converted into one another, facilitating inter-species comparisons. However, a significant drawback of this approach is that regions, which are not evolutionary conserved between the original and target species, are lost. It should be noted, that liftOver facilitates the conversion between genomic loci of different species or among different generations of genome assemblies, but not between different gene nomenclatures. Further tools for annotation transfer are discussed elsewhere [22].

### Visualization of sequencing data

A helpful step to gain an initial impression of sequencing data or to view specific genomic regions in detail is to visualize genome-wide sequences relative to the reference genome. This can be achieved by tools such as the UCSC Genome Browser or Integrative Genome Viewer (IGV) [21, 31]. Uploading sequencing data to either website will allow users to graphically visualize genomic data, search them for gene names and genomic coordinates, and compare multiple datasets. Alongside the uploaded data, additional pre-installed genomic information is provided in both tools such as ChIP-seq data for histone modifications or common transcription factors, SNPs, conservation across species or repeating elements. Yet, while UCSC Genome Browser outperforms IGV in the availability of additional datasets, the graphical display and image quality is superior in IGV as here content can be directly exported as vector graphics. UCSC Genome Browser on the other hand provides *.eps* graphics, which can be converted into publication-quality figures using appropriate software such as INKSCAPE.

### Software choice

Most tools discussed in this review are available as graphic-user interfaces (GUI) such as online or desktop versions and command line-run programs. For inexperienced users, GUI versions may be a good choice, as these usually provide intuitive handling and easier navigation. To target our discussion to wet-lab based researchers who may have little to no prior experience with bioinformatic computing, we will focus on tools that are available online as these come without installation requirements. However, to make the most of the application possibilities of a given tool, it may be advantageous to resort to desktop or even command line versions, as for many tools these include more customization options and can run faster than online distributions. For most of the tools presented here, online tutorials of their application are provided on the respective websites. Users who are interested in diving deeper into the bioinformatic application of these tools are advised to become familiar with Unix command line navigation, as well as programming in R and Python.

## Assessment of protein/DNA interaction: ChIP-seq

Epigenetic modification of chromatin together with the temporally and spatially controlled contact of TFs and their transcriptional co-regulators lie at the core of gene expression regulation. A variety of techniques has been developed in recent years to map the occupancy of TFs and histones on DNA and detect the chemical modifications these carry. One of the first and still the most widely used method to assess the chromatin landscape genome-wide is chromatin immunoprecipitation (ChIP) [54] followed by massive parallel sequencing (ChIP-seq) [3]. Briefly, ChIP uses polymerization of paraformaldehyde (PFA) to crosslink proteins to chromatin. After cell lysis and recovery of the cell nuclei, the chromatin is fragmented by sonication or micrococcal nuclease digestion. The fragmented chromatin is then precipitated with antibodies directed against the TF or histone modification of interest. Protein-DNA complexes are recovered, washed to reduce background signals and the precipitated DNA is isolated by heat-induced crosslinking reversal. The DNA fragments are then subjected to library preparation and, after indexing and quality control (QC), samples are sequenced using an appropriate next-generation sequencing platform. Following a series of QC steps (which include eliminating contaminating DNA sequences from other commonly used model organisms using FastQC [29], removing remaining adapter sequences, and quality trimming), the reads are mapped against an appropriate reference genome. Mapped reads are then filtered to retain only high confidence concordant pairs, usually followed by the removal of reads mapping to the mitochondrial genome and unassembled contigs. Peak calling is performed, and candidate regions are further filtered by fold enrichment score. In this stage of analysis, datasets are most commonly deposited in public repositories. Different peak calling algorithms are in use. While TF binding sites are usually called assuming narrow peaks, for histone modifications broad peak callers are employed. When using data from the public domain, it should always be cross-checked with other publications whether peak width of a certain dataset is in the appropriate range for the assessed entity. Performing some of these simple but effective quality control methods can be of great help, especially when working with data that originate from the public domain.

### Databases

One useful public repository for retrieving datasets is ChIP-Atlas, a fully integrated data-mining suite for ChIP-seq, DNAse-seq, ATAC-seq, and Bisulfite-seq data [6, 7]. This database serves the assembly of datasets from various sources and organisms, including human and mouse. It shows alignment and peak-call results in several formats including *.bed* as well as *.bigWig* for ChIP-seq data. Alongside data retrieval, ChIP-Atlas allows analyzing genome-wide transcriptional regulator interactions with one another or with genes of interest, as well as examining enrichment of protein binding for multiple genomic coordinates or gene names. In addition, ChIP-Atlas offers options to visually assess the quality of different types of sequencing data, a requirement for any meaningful further analysis. The representation of ‘Base call quality data from DBCLS SRA’ in ChIP-Atlas allows to visually determine data quality in the form of a homogeneous distribution of quality scores spanning the green area of QC plots. Another database harboring human and murine data from ChIP-seq, DNase-seq and ATAC-seq experiments, which can be used to extract further cis-regulatory information, is Cistrome DB [8, 55]. While fewer datasets are available on Cistrome DB than ChIP-Atlas, additional functions are implemented, such as QC and motif discovery, which is a clear advantage of this database. ChIPBase is a third possibility to collect datasets, enabling direct performance of motif discovery [9, 10]. While this database focuses on the function of non-coding RNA (ncRNA) entities, ChIP-function can be initially assessed by correlation with expression of TFs as indicated by RNA-seq. A drawback of ChIPBase is that raw peak data cannot be downloaded, but a reference to GEO Accession Viewer is provided, through which access to the original data is possible. Finally, large-scale integrative analysis can also be performed with ReMap, another collection of manually curated ChIP-seq, ChIP-exo, and DAP-seq (DNA Affinity Purification Sequencing) data from public sources (GEO, ENCODE, ENA) [11].

**Fig. 2:**
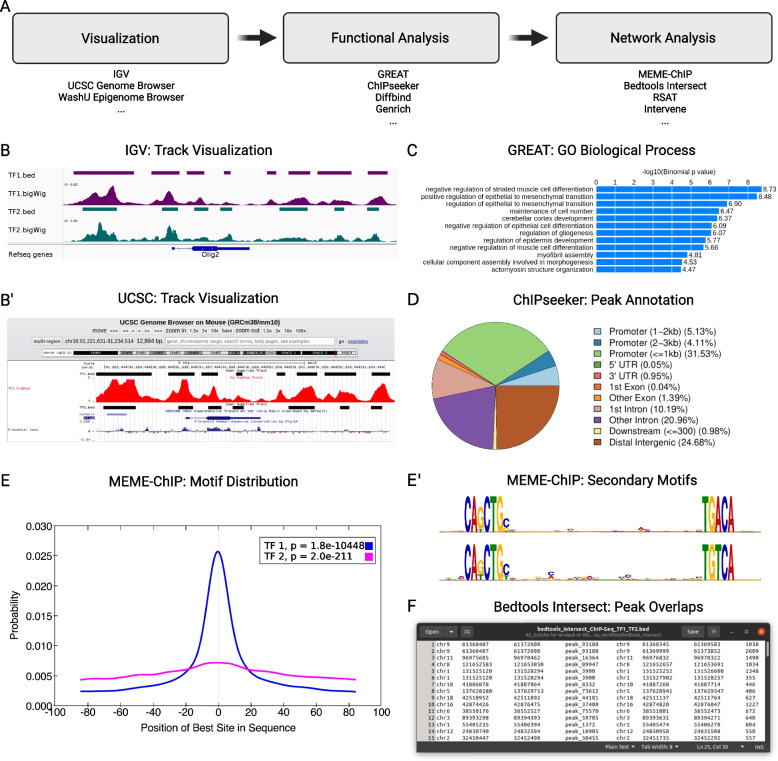
Exemplary ChIP-seq analysis pipeline and outputs. **(A)** Exemplary workflow and suggested tools, **(B)** overlay of ChIP-seq tracks in IGV and **(B’)** UCSC Genome Browser, **(C)** associated GO terms of ChIP-seq data obtained by analysis with GREAT, **(D)** genomic annotation of ChIP-seq peaks with ChIPseeker, **(E)** motif distribution of two exemplary TFs in whole genome and **(E’)** exemplary secondary motif spacing derived from MEME-ChIP analysis, and **(F)** dataset intersection of two exemplary TFs using bedtools intersect, visualized with simple text editor program (columns 1-4: peak information TF1 [peak chromosome, start, stop, name], columns 5-7: peak information TF2 [peak chromosome, start, stop], column 8: overlapping peak width)

Downloaded ChIP-seq datasets can then be subjected to post-analysis and in silico assessments by a specific workflow that we present below. A schematic of this workflow is summarized in Fig. 2 A, and exemplary outputs are displayed in Fig. 2 B-F. For simplicity, only one possible approach is described below, in which we focus on the identification of regulatory interactions in chromatin. However, many different analysis routes are possible and, depending on the initial data structure, other approaches than the ones detailed below may be suitable. Figure 1 lists several tools and platforms, together with their respective input data formats and purposes that can be used on ChIP-seq data. Table 2 gives an overview over some of the most prominent tools that can be used instead or in addition to those discussed below.

### Visualization

As described above for the visualization of general sequencing information, called peaks and sequencing tracks generated in the course of ChIP-seq experiments are commonly visualized in genome browsers relative to a reference genome and relevant genomic features. The two most common formats for ChIP-seq data are *.bed* for called peaks and *.bigWig* for continuous sequencing tracks. There are several genome browsers to choose from, depending on the origin of the data one would like to visualize. ChIP-Atlas can be easily combined with IGV (Fig. 2 B), while Cistrome DB carries direct plugins for UCSC Genome Browser (Fig. 2 B’) [21, 30] and WashU Epigenome Browser [32]. Data curated in ReMap as well as ENCODE can be directly accessed via UCSC Genome Browser and multiple factors can be integrated for parallel visualization.

### Functional analysis

A useful next step is to assess ChIP-seq datasets in terms of potential biological functions. The Genomic Regions Enrichment of Annotations Tool (GREAT) is a good choice for predicting functions of cis-regulatory regions [26]. Any set of genomic regions in *.bed* format can serve as input to this GO term analysis tool. However it should be noted, that the current version of GREAT only supports human (hg19 and hg38) and mouse (mm9 and mm10) assemblies, and data from different species or assemblies need to be converted first using liftOver. Outputs can be visualized either as bar chart or interactive ontological hierarchy (Fig. 2 C). Additionally, ChIP-seq peaks can be subjected to peak annotation and visualization with ChIPseeker (Fig. 2 D) to gain a deeper understanding of where peaks are localized relative to distinct genomic sites such as promotor regions, and intragenic or intergenic genomic sequences [20]. While GREAT needs to be accessed through the respective website, ChIPseeker can be used via the platform Galaxy. To this end, a *.gtf* file harboring the corresponding genome assembly (e.g. comprehensive gene annotation) needs to be retrieved from GENCODE [56], and uploaded to Galaxy. Galaxy offers many additional functional analysis tools, such as DiffBind for differential binding analysis of ChIP-seq data [57], or Genrich to detect sites of genomic enrichment. Tools available on this platform are easily explored, as they come with a comprehensive overview of their features, and supported input and output formats.

### Network analysis

A frequently used method to better understand the underlying logic of a given transcriptional regulation scheme is to assess the regulatory network in the form of motif discovery using MEME-ChIP. This tool takes its input in *.fasta* format. However, *.fasta* format is not provided by many platforms but can be re-constructed on basis of more common *.bed* formats with the help of the getfasta function in the bedtools toolkit (available on Galaxy). MEME-ChIP is designed for the analysis of ChIP-seq ’peak regions’ [27, 58]. These expected binding regions are defined as short genomic sequences of 6-12 bp in length surrounding the summit of ChIP-seq peaks, i.e. the individual local maxima of alignment reads in a given ChIP-seq experiment (e.g. the TF binding site in case of a ChIP-seq experiment for a TF). Given a set of genomic regions, MEME-ChIP performs a series of *ab initio* analyses, such as primary and secondary motif discovery, motif distribution, motif enrichment analysis, motif visualization, binding affinity analysis, and motif identification (Fig. 2 E). Moreover, datasets can be subjected to spaced motif analysis (SpaMo), which infers physical interactions between a previously defined TF and TFs bound at neighboring sites at the DNA interface, whereby close proximity of TF motifs indicates potential interaction (Fig. 2 E’) [59, 60]. Another platform to perform *de novo* motif discovery or motif scanning to predict TF binding sites is RSAT. While RSAT operates similarly to MEME-ChIP, it integrates more database options for motif discovery. Furthermore, RSAT includes original analysis, such as motif quality evaluation, motif comparisons and clustering, detection and analysis of regulatory variants, building of control datasets, and comparative genomics to discover motifs based on cross-species conservation [28].

Public repositories can also be searched for datasets of such factors for which potential interaction functions are indicated by motif analysis. Potential co-binding can be assessed by overlap computation of ChIP-seq peaks in *.bed* format using the bedtools intersect function [25] available on Galaxy. This tool generally enables genome arithmetic and can be used to merge, count, complement, and shuffle genomic intervals from multiple files in widely used file formats (an exemplary intersection output of two TF ChIP-seq datasets is shown in Fig. 2 F). Alternatively, the intersection tool Intervene available on Galaxy can be applied, which allows to produce Upset plots of multiple intersections [61].

### Alternatives to ChIP-seq

Despite its experimental power and wide application, ChIP-seq remains challenging with small samples and binding sites can be mapped only within 100-200 base pairs, limiting the resolution of this method. In ChIP-exo, this problem is alleviated by including a trimming step of the precipitated DNA fragments by lambda exonucleases [62]. In ChIP-chip (ChIP-on-chip), DNA fragments are isolated by ChIP and assessed by hybridization to genomic microarrays [63]. Both methods have found less widespread use than ChIP-seq, but data generated by them can be examined similarly to the analysis pipelines described above for data generated by ChIP-seq if appropriate data formats are available. A further limitation of ChIP is the reliance on highly specific antibodies that recognize their target after formalin-fixation of the chromatin. As a solution to this problem, DamID (DNA adenine methyltransferase identification) offers an approach to identify target sites of chromatin-binding proteins on the genome without the need to have suitable antibodies available. Instead, the DNA-binding protein is ectopically expressed as a fusion to E.coli DNA adenine methyltransferase [64]. Sequencing data generated by DamID can be assessed by the tools described above for ChIP-seq, although specialized tools are available for the initial steps of the data processing workflow such as sequence alignment or read extension. A detailed pipeline can be found on GitHub.

Two relatively new technical improvements for chromatin profiling that are becoming increasingly popular are CUT&RUN (Cleavage Under Targets and Release Using Nuclease; [65]) and CUT&Tag (Cleavage Under Targets and TAGmentation; [66]). Both techniques rely on the fusion of protein A, required for the purification of antibody-precipitated DNA, to a DNA-cleaving enzyme, micrococcal nuclease (MNase) in CUT&RUN or Tn5 transposase in CUT&Tag. Both approaches offer an improved signal to noise ratio compared to ChIP-seq, making them better suited for low cell numbers. Unlike ChIP or DamID, CUT&RUN and CUT&Tag are performed on unfixed cells and therefore not affected by possible fixation-induced artefacts. A pipeline for analysis and visualization of CUT&RUN and CUT&Tag data is provided by CUT&RUNTools [67]. However, for its application one has to delve a little deeper into bioinformatics as currently no web-based analysis tool is available. Navigation through GitHub alongside some previous experience with Python code are therefore required to apply this toolkit. For pre-analyzed CUT&RUN and CUT&Tag data, the GEO Accession Viewer again provides datasets for several biological contexts and transcriptional regulators.

## Assessment of chromatin accessibility: ATAC-seq

Condensed chromatin, characterized by packaging with linker histone H1 and tight DNA wrapping around nucleosomes, prevails in transcriptionally inactive regions, while open chromatin regions, i.e. stretches of DNA exhibiting depletion of nucleosomes, are associated with transcriptional activity [68, 69]. Mapping genome-wide changes in chromatin accessibility has thus long served as a way to identify regulatory elements and study the relationship between chromatin structure and transcriptional activation. Different NGS-based paradigms for epigenetic profiling of open chromatin and nucleosome positions have been developed: DNase-seq (DNase I hypersensitive sites followed by massive parallel sequencing) uses the endonuclease DNase to cleave DNA within accessible chromatin, followed by library preparation and NGS [70]. MNase-seq uses the endonuclease/exonuclease Micrococcal nuclease (MNase) to eliminate accessible DNA and selectively sequences nucleosome-bound DNA [71]. FAIRE (Formaldehyde-Assisted Isolation of Regulatory Elements) sequencing involves formaldehyde cross-linking of proteins to DNA, shearing of the DNA, recovery of the nucleosome-free DNA-fragments by phenol-chloroform extraction, and NGS [72]. In the Assay for Transposase-Accessible Chromatin using sequencing (ATAC-seq), hyperactive Tn5 transposase integrates into open chromatin regions where it simultaneously cuts and ligates adapters for library preparation and high-throughput sequencing [2, 73]. This underlying principle allowed ATAC-seq to be developed further to include methods to create chromatin accessibility maps of individual cells [74, 75]. Irrespective of the NGS-based technology that was used to profile chromatin accessibility, open chromatin regions can be annotated bioinformatically, and post-hoc analysis such as DNA-footprinting or analysis of motif enrichment (AME) can be performed. For a further discussion, the reader is referred to [4].

Because ChIP-seq and ATAC-seq both yield partial genome reads annotated to the whole genome as results, the tools described above for analysis of ChIP-seq results can also be applied to ATAC-seq analysis. Further, data generated by ATAC-seq and ChIP-seq experiments can be combined in multiple ways, and ATAC-seq datasets can also be retrieved through ChIP-Atlas. For ATAC-seq, some simple forms of quality control can be applied. For example, transcription start sites (TSS) of actively transcribed genes always have a more open chromatin environment, so ATAC-seq data should inevitably contain TSS. Starting from ATAC-seq results, and thus from genomic regions that classify as ’open’ in a particular cell population or tissue, motif discovery can be applied to determine which TF binding motifs these sequences harbor. This approach will give a first indication of the types of TFs that can bind to these genomic regions, in principle. ChIP-seq data for these TFs in the same or related cells and tissues may then be retrieved from the public domain and compared one by one to the initial ATAC-seq results. This can be done with the help of tools like the already described bedtools intersect to narrow down the list of candidate TFs involved in gene expression regulation through the genomic sequences identified in the initial ATAC-seq experiment. If ChIP-seq data for TFs of interest are not available, in silico analysis of ATAC-seq can precede ChIP-seq experiments. In such cases, promising TF candidates for immunoprecipitation may be identified by motif analysis of open chromatin regions with help of MEME-ChIP or RSAT, followed by assessment of the corresponding TF-DNA binding by ChIP-seq experiments in the laboratory.

## Assessment of gene activity: RNA-seq

The most commonly used high-throughput technique in transcriptomics is bulk RNA-sequencing (RNA-seq). It provides insight into the transcriptome of tissue sections, biopsies, or cell populations. Although further methods, that will be discussed below, have been developed in the recent years and despite the caveat that bulk RNA-seq determines the average expression level of individual genes over a large and frequently inhomogeneous starting cell population, bulk RNA-seq also has considerable strengths as compared to alternative approaches. The focus of bulk RNA-seq is on global changes in the transcriptional profile. Major advantages of bulk RNA-seq are the easy application and relatively low prices, providing better accessibility compared to single-cell RNA-seq (scRNA-seq), in which an assessment of heterogeneity is the focus. For these reasons, bulk RNA-seq datasets are frequent in the public domain. However, both methods have their limitations. scRNA-seq is more cost-intensive, suffers from cell dropout and reduced coverage of genes and physiologically occurring fluctuations in expression are often overrepresented. Bulk RNA-seq on the other hand measures gene expression in mixtures of cells and, consequently, cannot distinguish between low-abundant transcripts in large cell populations and high-abundant transcripts in small populations. It will be the focus of this chapter to present tools for the in-depth analysis of bulk RNA-seq datasets, which non-specialists can make use of. Nonetheless, the involvement of a trained bioinformatician is certainly highly recommended to fully evaluate sequencing data quality and as support to learn and apply the tools presented in this paper. In addition, even the best and most sophisticated analysis approaches cannot compensate for low quality data and a bioinformatician can point out the limitations of the original data. Which approach is the right one depends on the question at hand and is up to the investigator to determine. In addition, when making use of public datasets or analyzing their own datasets, users are recommended to critically assess the study outline under which the data was generated, whether homogeneity of the sample was ensured, and appropriate control experiments were executed for the reported claims. Specifically, we recommend making sure that the expected outcomes of the dataset, for example a transgene expression profile, have been satisfied and the data quality metrics are acceptable. Again, we recommend taking the support of a trained bioinformatician if needed for this crucial initial aspect of the data analysis.

RNA-seq allows the analysis of protein-coding mRNAs and ncRNA such as ribosomal RNA (rRNA) or microRNA (miRNA). For this matter, high quality total RNA is extracted from cells or tissues. Different sub-populations of RNA can be enriched or depleted to increase sequencing depth. Ribodepletion, which removes the abundant rRNA but leaves the full diversity of other RNAs intact, is usually carried out as enrichment step. In cases where RNA subpopulations are in focus, other isolation protocols can be applied e.g. size selection for long ncRNAs or small ncRNAs, or poly-A enrichment to specifically enrich mRNA. Following the choice of RNA subsets, the RNA is converted to complementary DNA (cDNA) by reverse transcription and sequencing adaptors are added to one or both ends of the cDNA fragments. After amplification of the fragments, the RNA-seq library can be sequenced by various paradigms using NGS platforms [76, 77]. When performing RNA-seq, normalization of sequencing depth and gene length to permit comparison of results between genes and samples is obtained by one of three measures: Reads Per Kilobase Million (RPKM) for single-end RNA-seq, Fragments Per Kilobase Million (FPKM) for paired-end RNA-seq, or Transcripts Per Kilobase Million (TPM), which can be used for both sequencing paradigms. As of now, validation of RNA-seq experiments by qPCR is a standard in good experimental practice, and is a useful starting point when building hypotheses on public domain datasets. However, it must be noted that qPCR is a sensitive methodology for detecting relative levels of a particular transcript, whereas RNA-seq datasets are limited by their sequencing depth. This aspect can be appreciated particularly when comparing scRNA-seq with bulk RNA-seq as mentioned in the section comparing these two analyses types. Thus less abundant transcripts may be absent in sequencing datasets, while they are often detected by qPCR. Therefore validation of less abundant genes may not yield comparable results by the two methods. Whether this experimental practice will be uphold in the future, will depend among others on the abundance of available datasets on a given physiological context in the public domain.

### Platforms and databases

RNA-seq datasets can be accessed through various databases, including GEO Accession Viewer, R2 and ARCHS4 [78]. Available data formats on GEO vary greatly among datasets, ranging from spreadsheets over *.txt* to graphic formats such as *.bedgraph* or *.bigWig*, as no standardized upload criteria are defined for this repository. For direct analysis of GEO RNA-seq data, GEO2R may be used to perform differential expression analysis. The R2 Genomics Analysis and Visualization Platform is another option for exploring and analyzing gene expression data. It contains datasets from large numbers of array-type gene expression profiling studies together with bulk RNA-seq, scRNA-seq and some ChIP-seq datasets. Any public dataset can be added to the R2 platform upon request using the accession ID of the dataset. This aspect is similar to the GREIN platform which will be discussed below. The R2 platform allows users to explore gene expression data in multiple ways, including the correlation of genes (with other genes and with sample groups) and the analysis of differential expression between groups (by DESeq2 or other tests). Within the framework of R2, data can also be subjected to KEGG pathway analysis between groups or by correlation. R2 further provides the option to parametrically analyze gene set enrichment (PAGE) [79], to perform survival analysis (Kaplan-Meier) and gene onotology analysis for suitable datasets, and to create classic PCA plots, volcano plots, heatmaps, as well as Upset plots. For RNA-seq data, some databases do provide data quality information. For example the GREIN platform provides information about the sequence alignment scores, duplicate reads, sequence counts for each sample and indicates whether it passed the quality test or not. Likewise, RNA-seq data can be expected to be enriched in exonic sequences, and, hence, the overrepresentation of exonic sequences in RNA-seq data can be regarded as a sign of confidence. We strongly recommend users to make sure that the data quality is acceptable before in-depth analysis of a particular dataset. In case no quality information is provided, users are advised to check for expected expression profiles, i.e. whether appropriate housekeeping or marker genes for the given context are present, and how many genes have average counts above a given number, thereby ensuring a good statistical basis for differential gene expression analysis. A particular challenge for working with data from different sources, especially when the data comes from older studies, is the often ambiguous gene nomenclature. In the past, multiple alternative paradigms were developed for gene identification, resulting in many genes having been assigned multiple names. In such cases, it is up to the researchers themselves to identify alternative or redundant gene names. In this case tools for the conversion of common nomenclatures can be helpful such as BioTools.fr and g:Convert of the g:Profiler toolset [50].

**Fig. 3:**
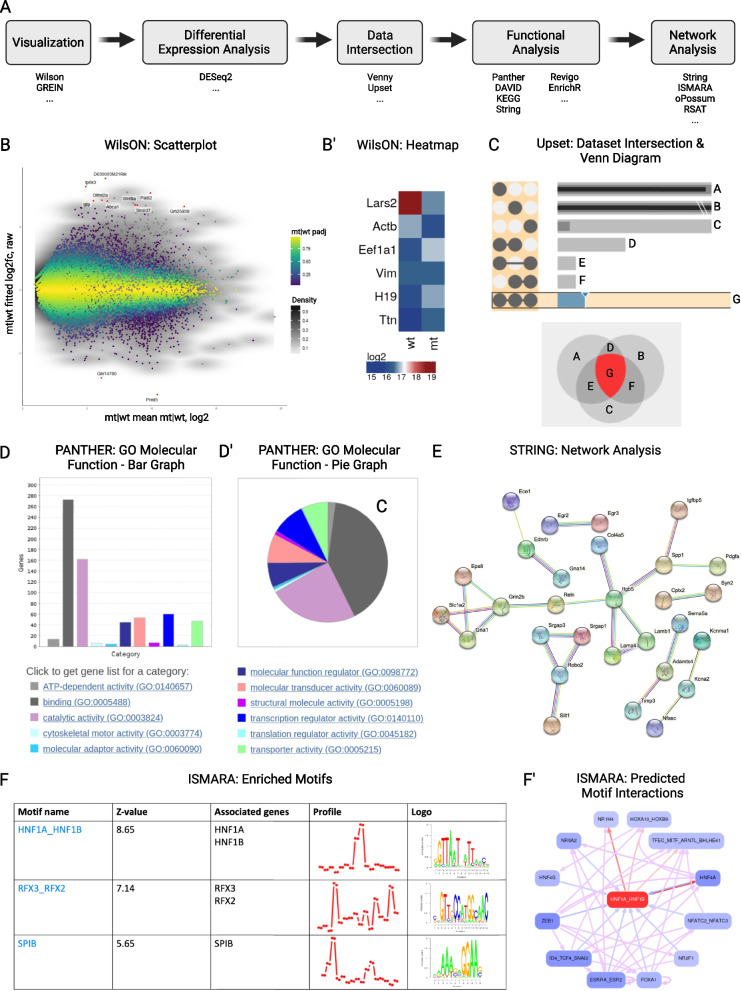
Exemplary RNA-seq analysis pipeline and outputs. **(A)** Exemplary workflow and suggested tools, **(B)** Scatterplot and **(B’)** heatmap obtained by WIlsON analysis, **(C)** visualization of dataset intersection in UpSet plot and venn diagram, **(D)** bar plot and **(D’)** pie diagram of GO terms obtained with PANTHER, **(E)** interaction network obtained by STRING analysis and **(F)** motif enrichment of differentially expressed genes and **(F´)** predicted motif interactions using ISMARA to assess potential transcriptional regulators

Below we present an analysis pipeline to make use of retrieved RNA-seq datasets from the public domain, following a certain workflow. A schematic of this workflow is exemplified in Fig. 3 A, and exemplary outputs are displayed in Fig. 3 B-F.

### Visualization

For visualization and analysis of RNA-seq data, a multitude of tools are available and are summarized in Table 3. Just as for ChIP-/ATAC-seq, sequencing tracks can be directly visualized using genome browsers. However, while genomic ChIP-/ATAC-seq data usually contain coding and regulatory regions of the genome, expression data lack that part of the genome that is not transcribed. For these differences in materiality of genomic and expression data, plain visualization of RNA-seq tracks, unlike the visualization of ChIP-/ATAC-seq tracks, is of limited explanatory power. Therefore, the use of other tools that highlight the distinct properties of RNA-seq data, such as differences in expression between conditions, is indicated. A useful tool to gain first insight into RNA-seq data, is WIlsON (Webbased Interactive Omics visualizatiON) [35]. WIlsON requires CLARION (generiC fiLe formAt foR quantItative cOmparsions of high throughput screeNs) files as input. This file format relies on a tab-delimited table with some metadata describing the columns that can be easily constructed from any tabular formats. It supports data that can be reduced to features (e.g. genes or transcripts) and their annotation with assigned numerical values (e.g. count or p-value). Those feature annotations and numerical values can later be used for filtering and plotting purposes, as exemplified in Fig. 3 B and B’. An original tab-delimited table (e.g. RNA-seq data) can be reformatted manually into a CLARION file using a spreadsheet software following the instructions in the WIlsON documentation. An example of RNA-seq data and its conversion to a CLARION file can be found in this GitHub folder. Once the CLARION file is loaded into the WIlsON app, users can generate various plots following four basic steps: (i) filtering for features, (ii) selection of plot type, (iii) adjusting plot parameters, and (iv) rendering/downloading results. Possible types of analysis include PCA, heatmap and scatterplot.

Another interactive web-based platform to explore and visualize RNA-seq data is GREIN [36]. The common features of WIlsON and GREIN include visualization to obtain metadata of the samples, counts tables and QC reports, correlation plots, PCA plots and heatmaps. A major difference between both platforms is that data can be highlighted and individual genes are searchable in WIlsON, while GREIN offers both 2D and 3D tSNE (t-distributed stochastic neighbor embedding) plots, in which high-dimensional data is reduced to minimal descriptive features and visualized. In addition, GREIN uses GEO IDs of existing public datasets as input, making it more user-friendly, whereas WIlsON relies on CLARION files. Both offer high quality data download options.

### Differential expression analysis

After an initial insight into the data, the standard analysis step for RNA-seq data is to determine differentially expressed genes between at least two treatment groups. Multiple tools are available for this purpose, but we will focus on DESeq2 as it is commonly used and available on the Galaxy platform [37]. DESeq2 is a popular statistical package written in R. It was initially developed to perform differential expression analysis of RNA-seq datasets, but is also applicable to comparative assessments of ChIP-Seq and mass-spectrometry results [37]. The statistical analysis is based on negative binomial linear models that are used to estimate the logarithmic fold changes and the strength of these changes considering inter-sample variation. Once count data are obtained after mapping the raw data (e.g. *.fastq* files) to the reference genome, the counts or reads for each gene and sample including its replicates can be analyzed by the DESeq2 package for differential expression. This approach yields both fold-changes and statistical significance for every expressed gene between the samples of interest. The DESeq2 package is included as a part of many RNA-seq pipelines and platforms (such as R2, WIlsON, or GREIN). Alternatively, DESeq2 can be directly implemented in R using the Bioconductor package as described by Love and colleagues [37].

### Data intersection

One of the most common bioinformatic analyses performed on -Omics data is the intersection of various datasets for shared features and differences. This has been classically done using Euler or Venn diagrams, the latter being available through several web-based analysis platforms such as Venny [33] or Galaxy [80, 81]. While the classic diagrams are easy to understand and interpret up to a small number of sets, they become more complicated when the number of sets increases to 4 or more. This is because the number of intersections (2$^n$) increases exponentially with the increase in number of sets (n). For the latter purpose, Lex and colleagues introduced UpSet, a matrix-based visualization of intersecting sets that is also amenable to visualization of the associated elements [34].

In order to visualize datasets on UpSet, an input file is created in Python or another supported programming language. For upload, two files are necessary. Firstly, a *.csv* spreadsheet comprising the binary data of the sets to be visualized, and secondly a *.json* file, an open standard language-independent file format that is used frequently for reading data from a server for use in online platforms. The *.json* file contains metadata of the *.csv* file as well as its location and name, and needs to be stored in an accessible location such as a shared folder on a public server or in a public repository such as GitHub. An example is available here, and can be used as template for the construction of *.json* and the corresponding *.csv* files [82]. To generate the *.csv* file, a binary data spreadsheet file has first to be created from the RNA-seq data. A simple binary transformation can be 1 for regulated genes and 0 for non-regulated genes between 2 conditions. Once the binary sets of interest are created, a *.csv* spreadsheet file should be set up with user defined headers for the binary data columns. Additional columns for fold changes or reads etc. can be included in this UpSet file for data visualization along with the intersection of sets with UpSet.

On the UpSet website, one needs to input the *.json* file created as described above. The genes comprising any particular intersection of interest can be visualized on the site under ‘Query Results’ after selecting a certain intersection on the UpSet plot (see Fig. 3 C). Specific genes of interest can also be searched in the ‘Query Filters’ menu. For the list of genes or elements displayed on the platform, a simple copy paste option allows the data import into a spreadsheet file (after selection of *.txt*). For UpSet plots, Venn diagrams, or any other features in display, only a screenshot option is available for storing the data.

### Functional analysis

Gene Ontology (GO) analysis is commonly executed following differential gene expression analysis to assess functions of genes and gene products. GOs are built as a transdisciplinary endeavor between Molecular Biology, Computer Science and Linguistics/Philosophy and as to the procedural progress of research, are continuously updated with the latest empirical evidence [83]. Different tools for GO term analysis exist, which build upon different logics, sources and gene concepts. Therefore, it is recommended to use multiple options for a deeper and more comprehensive understanding of the biological context under investigation.

One widely used GO analysis tool is PANTHER [40, 41]. This tool builds on a knowledge base curated by the Gene Ontology Consortium [84, 85]. PANTHER takes a list of gene names as input (supported are several ID-systems including Ensembl and Uniprot) and can work with a large number of different species. As output, the GO terms Molecular Function, Biological Process, Cellular Component, Protein Class, and Pathway are available (see Fig. 3 D and D´ for an exemplary analysis) and various statistical tests can be peformed. A full list of genes in the analyzed gene set that are associated with each pathway in the dropdown menu can be obtained via the associated hyperlink with each GO term. One drawback of PANTHER is the low quality of produced plots, but this can be bypassed by direct downloading of the data and plotting with R or any other data analysis platform of choice. Other GO term analysis tools include DAVID [44, 45], KEGG PATHWAY Database [46–48] and STRING [42, 43]. However, it should be noted that the main functionality of STRING is to provide information on protein-protein interactions of gene products as described below. Since the output of most of these tools are complex hierarchies of GO terms, another useful tool is REVIGO, which can be applied to reduce functional redundancy of GO term lists and visualize the results [49]. One downside of REVIGO is that it requires GO term IDs as input. Depending on the output format of the preceding GO term analysis step, it may become necessary to retrieve these IDs manually. Further discussion of the above described GO analysis tools can be found elsewhere [86].

Enrichr is another interactive and collaborative gene list enrichment analysis tool, which can be applied to various genomics data, including data obtained from ChIP-seq and ATAC-seq experiments [39]. The required input format for Enrichr are Entrez gene symbols. The program allows to query a given list of input gene symbols for various characteristics, such as consensus TFs, lncRNA, epigenetic roadmaps of histone marks, and various other enrichment paradigms that may be associated with these genes. In contrast to GO term analysis tools like PANTHER or DAVID, which perform population-based statistics and therefore perform more reliably on larger gene sets, Enrichr can be queried for any number of genes, even single genes.

### Network analysis

A frequent feature of transcription control is the reciprocal regulation of gene activities, including feedback- and feedforward-loops, both of which can be of highly complex dynamics and often operate in parallel. Such multi-factorial regulatory networks can be explored in silico with the help of computational approaches.

STRING (Search Tool for the Retrieval of Interacting Genes/Proteins) uses RNA-seq data to examine whether functional relationships may exist among gene products. STRING requires a list of gene names as input and performs network analysis on them, making use of the STRING database of known and predicted protein-protein interactions [42, 43]. A network of connected genes displayed as cloud of spheres and lines is given as output (see Fig. 3 E). This can be assessed interactively and subjected to clustering analysis. The graphical output may be customized in its visual appearance and downloaded in high image quality.

RNA-seq data can further be subjected to a reverse analysis of gene expression regulatory networks. The aim hereby is to project transcriptional regulators that may function as upstream regulators of genes that were identified as differentially expressed in a given RNA-seq experiment. One such approach is the web-based tool ISMARA (Integrated System for Motif Activity Response Analysis) [51]. It is designed to perform motif discovery and to predict key TFs and miRNAs, which may be critical for the changes in gene expression observed in a given experiment. For motif analysis, the tool only requires raw gene expression data as input (RNA-seq or microarray data) from a set of biological samples, uploaded as *.fastq* or *.bed/.bam/.sam* alignment files. These input data can be directly used for automatic processing and modelling, based on pre-calculated annotations of hundreds of regulatory sites of several mammalian genomes. Once the analysis is complete, ISMARA provides a table with the motive activities found in the samples, sorted by the significance score, which ISMARA assigns to each motif. Besides motif names and significance scores expressed as z-values, the output file also includes gene names of TFs associated with the motif, the activity profiles across samples, and the consensus binding sequence of TFs, termed logos (see Fig. 3 F). Each of the listed motifs is further linked to another separate results page, containing additional information. These include the top target genes known to be regulated by the motif, the target genes network according to the STRING database [42, 43], respective gene ontology analysis of various categories, as well as predicted direct regulatory interactions between this and other motifs (see Fig. 3 F’). All collected information together with high-resolution images can be downloaded from the website. Repeating ISMARA analyses with sample averaging emphasizes contrasts between sample groups (e.g. treated vs. non-treated). ISMARA thereby allows the annotation of replicates and calculates motif activity profiles that are averaged over these replicates and thus enables a simple initial analysis of possible regulatory networks. However, ISMARA predicts the TF motifs only based on proximal promotors. This feature can be a shortcoming of this tool, as many TFs predominantly bind to distal or intragenic control regions of gene expression, like enhancers, rather than to proximal promotors. Finally, ISMARA can also be applied to ChIP-seq data for motif discovery, similar to MEME-ChIP described earlier. Alternative transcription factor binding site analysis tools are oPOSSUM [52] and RSAT *network-interactions* [28].

### Further applications of RNA-seq

Bulk RNA-seq determines the average expression level of individual genes over a large and often inhomogeneous starting cell population. This approach can deliver a wide range of information in various experimental setups but may not be sufficient when cellular and spatial levels need to be considered. Spatial transcriptomics and scRNA-seq are two new, sophisticated methods that fill these gaps. scRNA-seq allows to read the transcriptome of individual cells in great depth and, thus, delivers information of gene expression with cellular precision. This technical advance has greatly changed how gene expression is studied in biology and biomedicine. The boom in this technology led to an exponential increase of available scRNA-seq datasets, the navigation through which can be challenging. The Human Cell Atlas project pursues the ambitious goal to map every cell type in the human body. A comprehensive, manually curated and searchable list of single-cell transcriptomics studies, indexed by publication and including meta-data such as cell source, type of analysis, and protocol used can be found here [87]. scRNA-seq datasets can be accessed through GEO Accession Viewer, but numerous other collections exist, with The Single Cell Expression Atlas hosted by EMBL-EBI or the Cell Types RNA-Seq Atlas of Allen Brain Institute, which contains transcriptomic information from mouse and human cortex, being just two examples of many. Upon publication, pre-analyzed scRNA-seq datasets are often made accessible via interactive web applications, frequently presented as visually appealing Shiny Apps. These can be employed for in-depth assessment of individual genes and cell cohorts but mostly must be accessed through a link given in the respective original publication. Readers specifically interested in bioinformatic analysis of scRNA-seq data are referred to the large number of excellent recent reviews on this topic, such as [88–91]. A web-based, manually curated catalogue of software tools for the analysis of scRNA-seq data is scRNA-tools database.

While the cell-to-cell heterogeneity in populations of cells is kept in scRNA-seq, spatial transcriptomics retains the spatial information of transcripts within tissues [92–94]. The method quickly expanded in the last few years to include applications to epigenome sequencing via chromatin state profiling [95] and to ATAC-sequencing [96]. The positional information is obtained via arrayed barcoded oligonucleotides that are hybridized to overlaid tissue specimen. These approaches also allow multimodal spatial profiling for example with antibody-based protein barcoding approaches in parallel to next-generation RNA-seq [97]. The tools and platforms described in the current review are also applicable to spatial -Omics datasets once appropriate transformation of the data and clustering is performed to separate the positional information from the sequencing data. For example, RNA-seq data obtained from spatial transcriptomics can be analyzed by DESeq2 for comparing the raw data (*.fastq*) from 2 regions of interest to obtain differential gene expression between them. In addition, spatial transcriptomics data can be explored with the help of tools like the 10x GenomicsLoupe Browser or several specialized software packages [98], many of which can be accessed through bio.tools but require more programming experience.

## Integrative data analysis

In order to maximize the opportunities for insight that computational analysis tools provide, it is often necessary to triangulate and integrate information from various sources. Several tools and pipelines can be employed to bioinformatically integrate data from various sequencing experiments. One useful tool to annotate ChIP-seq peaks with the two closest genes is RnaChipIntegrator. However, unlike most of the other tools described in this review RnaChipIntegrator requires some programming and command line experience. Using RNA-seq as prompt, the RSAT module *retrieve-sequences* allows to extract upstream, downstream or open reading frame sequences [99], while RSAT *retrieve-ensembl-seq* retrieves sequences of promotors or other specified features on-the-fly from Ensembl [28]. These promotor regions can be subjected to downstream motif analysis to discover potential TF binding sites using RSAT *network-interactions*, as well as overlapped with relevant ChIP-seq data using bedtools intersect. In cases where ATAC-seq data is available, intersection of promotors and TF occupancy can be further refined by information about chromatin accessibility, thus integrating data from three different sequencing paradigms. Subsequently, after conversion of the peak data to gene sets e.g. following this Galaxytutorial, datasets can be subjected to STRING and thus interrogated for an in silico prediction of protein-protein interactions. Finally, once the above strategies have revealed genomic binding of one or more DNA-binding proteins in close proximity, proteomics databases such as PRIDE (PRoteomics IDEntifications Database) or BioGRID can be interrogated to determine whether corresponding protein-protein interactions have already been detected in similar biological systems [100–102].

## Conclusions

Traditionally, the epistemic culture in Molecular Biology used to follow an unidirectional path from hypothesis to data acquisition [103, 104]. In the post-genomics era, Biology has been increasingly informed by informatics as to cope with large-scale datasets produced by whole-genome sequencing approaches. Bioinformatics has since evolved as a subdiscipline of Molecular Biology, but the two research disciplines still need to be more fully integrated.

In this review, we present general resources and an exemplary analysis pipeline that integrates publicly available data types and multiple research methodologies. The use of published genomics data together with multi-layered data integration may constitute a new epistemic practice to uncover biological functions as well as their relationality in space and time. As an added benefit, resources may be used more sustainably, as new hypotheses can be first tested in silico before moving to experiments in the wet-lab. Indeed, in recent years an increasing number of researchers have integrated their own results with public datasets and used bioinformatic tools in their analysis similar to what we proposed in this review. This includes such broad applications as cellular senescence [105], carcinogenesis [38, 106], or immunology [107, 108].

Still, this approach necessitates a reciprocal reflection of the object of inquiry and the methodology used, and questions such as the following should therefore be asked: What kind of data are available and which data might be lacking to complement the picture? How was the data produced, what bias may have been introduced? Can biological contexts be compared (e.g. because of evolutionary relation) or should they be considered separately? What are relevant and ontologically meaningful controls? The latter point is particularly important when multiple datasets are compared, and corrections for multiple testing need to be applied. Because bioinformatic tools are continuously developed and new genomics datasets become available, the approach presented here must be considered as a procedural activity constantly under flux rather than a fixed pipeline. Experimental validation of bioinformatically derived hypothesis and in silico predictions should be triangulated with in vitro and in vivo approaches to bridge the gap of disciplinary languages, and to gain a deeper insight into the objects of inquiry in both material and informational dimensions.

## Data Availability

Materials provided in this manuscript are deposited on Github: $\bullet$ *.json* example: https://github.com/SGD2020/mcao $\bullet$ *.CLARION* example: https://github.com/veritasnondatur/Bioinformatics-for-Wet-lab-Scientists-Practical-Application-in-Sequencing-Analysis

## Abbreviations

AME: Analysis of Motif Enrichment
ATAC-seq: Assay for Transposase-Accessible Chromatin using sequencing
ChIP-seq: Chromatin Immunoprecipitation followed by sequencing
cDNA: complementary DNA
CLARION: generiC fiLe formAt foR quantItative cOmparsions of high throughput screeNs
CUT&RUN: Cleavage Under Targets and Release Using Nuclease
CUT&Tag: Cleavage Under Targets and TAGmentation
DamID: DNA adenine methyltransferase IDentification
DNase-seq: DNase I hypersensitive sites followed by sequencing
EMBL-EBI: European Molecular Biology Laboratory-European Bioinformatics Institute
ENCODE: Encyclopedia of DNA Elements
FAIRE: Formaldehyde-Assisted Isolation of Regulatory Elements
GO: Gene Ontology
GEO: Gene Expression Omnibus
GREAT: Genomic Regions Enrichment of Annotations Tool
GUI: Graphic-User Interface
IGV: Integrative Genome Viewer
ISMARA: Integrated System for Motif Activity Response Analysis
miRNA: microRNA
MNase: Micrococcal Nuclease
NCBI: National Center for Biotechnology Information
ncRNA: non-coding RNA
NGS: Next-Generation Sequencing
PAGE: Parametric Analysis of Gene set Enrichment
PCA: Principal Component Analysis
PFA: paraformaldehyde
PRIDE: PRoteomics IDEntifications Database
QC: Quality Control
rRNA: ribosomal RNA
RNA-seq: RNA-sequencing
scRNA-seq: single-cell RNA-seq
SpaMo: Spaced Motif analysis
STRING: Search Tool for the Retrieval of Interacting Genes/proteins
SRA: Sequence Read Archive
TF: Transcription Factor
tSNE: t-distributed stochastic neighbor embedding
WIlsON: Webbased Interactive Omics visualizatiON
